# Supplementary material for: ELAC2 Functions as a Key Gene in the Early Development of Placental Formation Based on WGCNA
Source: Cells. 2023 Feb 14;12(4):613. doi: 10.3390/cells12040613 (PMC9954566; doi:10.3390/cells12040613)
Supplement: Supplementary file 1 [file cells-12-00613-s001.zip › cells-2110392-supplementary.pdf]

A.Detection of ELAC2 in HTR-8/Svneo cells of different treatment groups

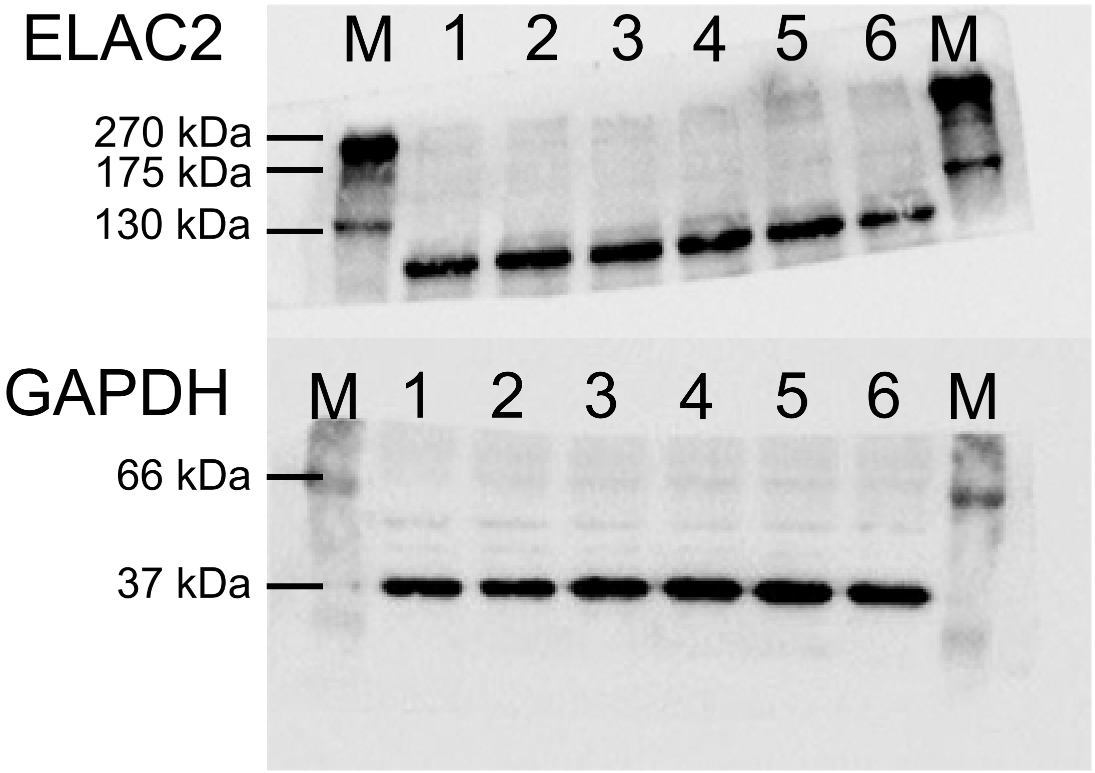

M:Marker

1,4: Blank

2,5: Negative control

3,6: si-ELAC2

B.Detection of Vimentin in HTR-8/Svneo cells of different treatment groups

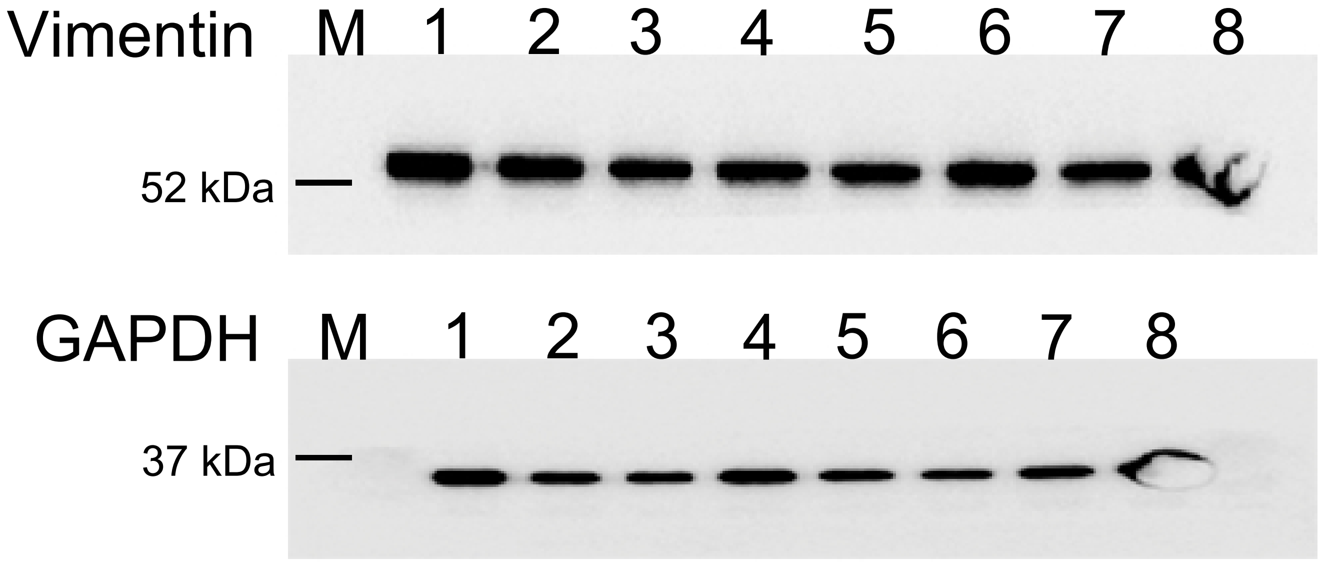

M:Marker

1,8: Blank

2,3,5,6: si-ELAC2

4,7: Negative control

C.Detection of E-cadherin in HTR-8/Svneo cells of different treatment groups

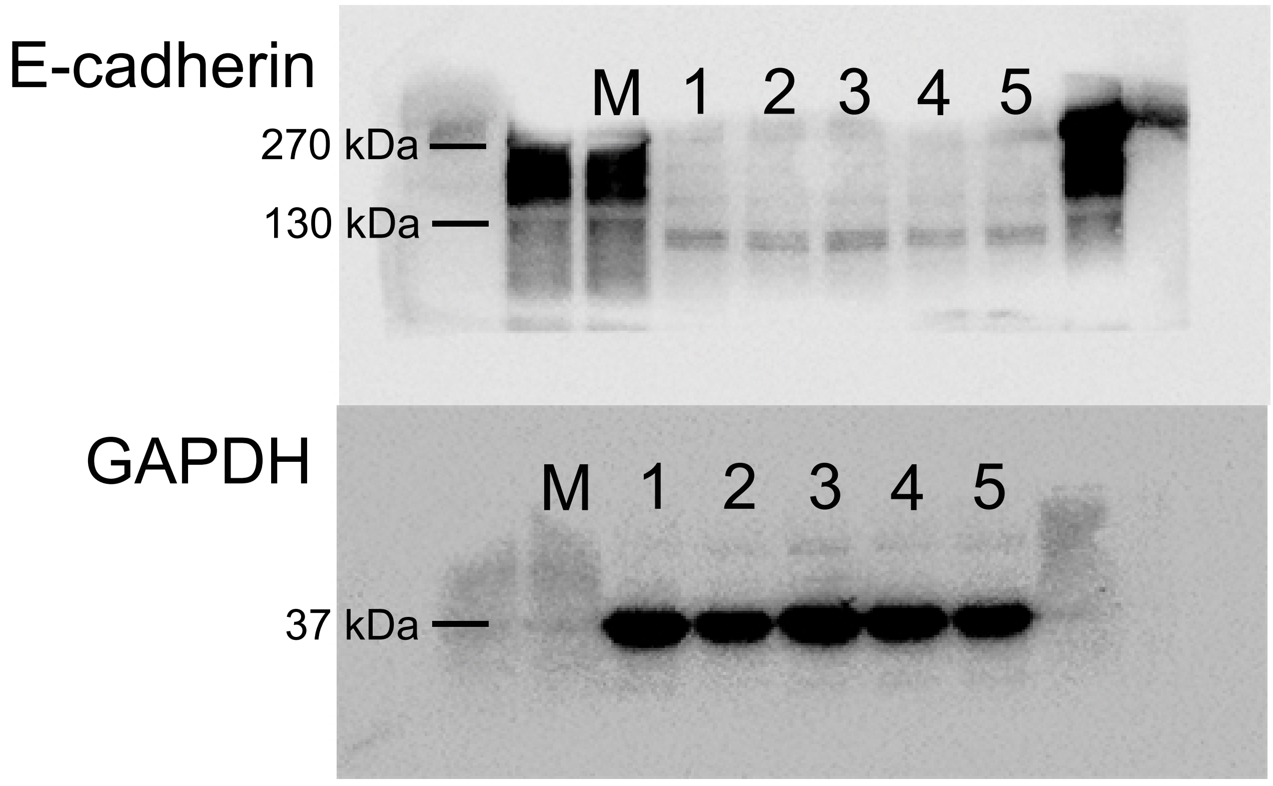

M:Marker

1,3: Negative control

2,4: si-ELAC2

5: Blank
